# Parental Patterns of Alcohol Consumption During the COVID-19 Pandemic: Scoping Review

**DOI:** 10.2196/48339

**Published:** 2024-08-26

**Authors:** Christine Ou, Kathryn Corby, Kelsey Booth, Hui-Hui Ou

**Affiliations:** 1 Canadian Institute for Substance Use Research School of Nursing University of Victoria Victoria, BC Canada; 2 School of Nursing University of Victoria Victoria, BC Canada; 3 Library Services University of the Fraser Valley Abbotsford, BC Canada

**Keywords:** parent, alcohol use, COVID-19, scoping review, parenting, alcohol, addict, addiction, substance use, health behavior, health behaviors, scoping, review methods, review methodology, drink, drinking, alcoholic, alcoholism

## Abstract

**Background:**

The declaration of the COVID-19 pandemic led to public health restrictions that impacted the lives of people across the globe. Parents were particularly burdened with balancing multiple responsibilities, such as working from home while caring for and educating their children. Alcohol use among parents is an area that warrants further exploration.

**Objective:**

This study aimed to investigate patterns of parental alcohol consumption during the COVID-19 pandemic, focusing on relative changes in the frequency and quantity of alcohol use compared to prepandemic use, nonparent adult samples, or both.

**Methods:**

A scoping review informed by the methodology of Arksey and O’Malley explored patterns of parental alcohol consumption during the COVID-19 pandemic. Searches were conducted in CINAHL, Ovid MEDLINE, PsycINFO, and Web of Science. Search terms were created using the Joanna Briggs Institute framework of Population, Concept, and Context, with the population being parents and the concept being alcohol consumption during the COVID-19 pandemic.

**Results:**

The database search yielded 3568 articles, which were screened for eligibility. Of the 3568 articles, 40 (1.12%) met the inclusion criteria and were included in the scoping review. Findings indicated the following: (1) having children at home was a factor associated with parental patterns of alcohol use; (2) mixed findings regarding gender-related patterns of alcohol consumption; and (3) linkages between parental patterns of alcohol use and mental health symptoms of stress, depression, and anxiety.

**Conclusions:**

This scoping review revealed heterogeneous patterns in parental alcohol use across sociocultural contexts during the COVID-19 pandemic. Given the known harms of alcohol use, it is worthwhile for clinicians to assess parental drinking patterns and initiate conversations regarding moderation in alcohol use.

## Introduction

### Background

On March 11, 2020, the World Health Organization (WHO) declared COVID-19 a global pandemic [1]. The restrictive public health measures introduced in many countries contributed to a shadow pandemic of psychological distress [2], which was associated with increased sales and consumption of alcohol [3,4]. Changes in the environments and circumstances in which adults drink can have effects on rates of consumption; lockdown restrictions and sheltering in place led to drinking in the home environment becoming the norm during COVID-19 lockdowns in some places, such as the United Kingdom [5].

In Westernized countries, problematic alcohol use peaks in the third decade of life, a time when many adults are raising young children [6]. The reduction of alcohol consumption is one of the top 10 modifiable risk factors for reducing disease burden, injury, and social problems globally [7,8]. The burden of disease associated with alcohol use is high. A meta-analysis identified alcohol consumption as the seventh leading risk factor for disability and premature death in 2016, and among those aged 15 to 49 years, alcohol consumption accounts for nearly 10% of deaths on a global scale [9].

Parents warrant special attention as a large subsection of the adult population because they are primary caregivers for children. For parents, pandemic stressors (eg, lockdown restrictions, balancing employment while children are at home, and reduced social support) compound the daily stressors of parenting young children [10]. In parallel to the elevated rates of parental depression and anxiety from prepandemic levels [11,12], evidence suggests that the COVID-19 pandemic has increased the consumption of alcohol in parents with young children [13,14]. A meta-analysis of 128 studies (aggregate sample of N=492,235) revealed that nearly a quarter of adults reported increases in alcohol consumption during the COVID-19 pandemic [15]. These changes were moderated by per capita gross domestic product and country. The authors identified that residing with children was associated with increases in alcohol consumption, with consumption increasing with the number of children at home [15]. Moreover, while Acuff et al [15] identified that female participants were more likely to increase their drinking frequency and male participants were more likely to increase their problematic drinking behaviors (eg, binge drinking), it is unclear what proportions of these participants were also caregivers to young children. Currently, a granular and gendered examination of patterns of alcohol consumption in caregiving adults is lacking during the COVID-19 pandemic.

### Objectives

The purpose of this scoping review was to investigate broad patterns of parental alcohol consumption during the COVID-19 pandemic, examining the relative changes in the frequency and quantity of alcohol use compared to nonparent adults, prepandemic levels of consumption, or both. In addition to physical health harms related to excessive alcohol consumption [16], parents who consume problematic amounts of alcohol are more likely to have worse mental health and lower emotional availability to children [13]. Poor parental mental health, substance use, and negative parenting practices can have adverse consequences on children’s socioemotional development and mental health [17,18]. The results of this scoping review can assist clinicians working with families in identifying parents at risk for alcohol misuse and engaging them in interventions to reduce consumption. The findings can also inform policy makers regarding the population of parents who may require targeted intervention and education on reducing and managing alcohol consumption in the aftermath of the COVID-19 pandemic.

## Methods

### Scoping Review Search Strategy

We undertook a scoping review following the methodology outlined by Arksey and O’Malley [19]. We used the Joanna Briggs Institute framework of Population, Concept, and Context (PCC) to create our search terms, with the population being parents (caregiving adults living with children aged <18 years in the same household) and the core concept being alcohol consumption within the context of the COVID-19 pandemic. To capture a wide scope of relevant studies, a librarian team member conducted a systematic search in CINAHL, Ovid MEDLINE, PsycINFO, Web of Science, and Cochrane databases using the specified PCC terms for all articles published after the WHO declaration of the COVID-19 pandemic on March 11, 2020 (Multimedia Appendix 1). The 3 groups of keywords within the PCC framework were joined with the Boolean operator “AND,” which produced 3568 articles for screening in June 2022. Medical Subject Headings and its descriptors were used for CINAHL, Ovid MEDLINE, and PsycINFO. Forward citation searching of articles identified in June 2022 was carried out in May 2023, which further yielded an additional 13 articles.

### Inclusion and Exclusion Criteria

**Figure 1:**
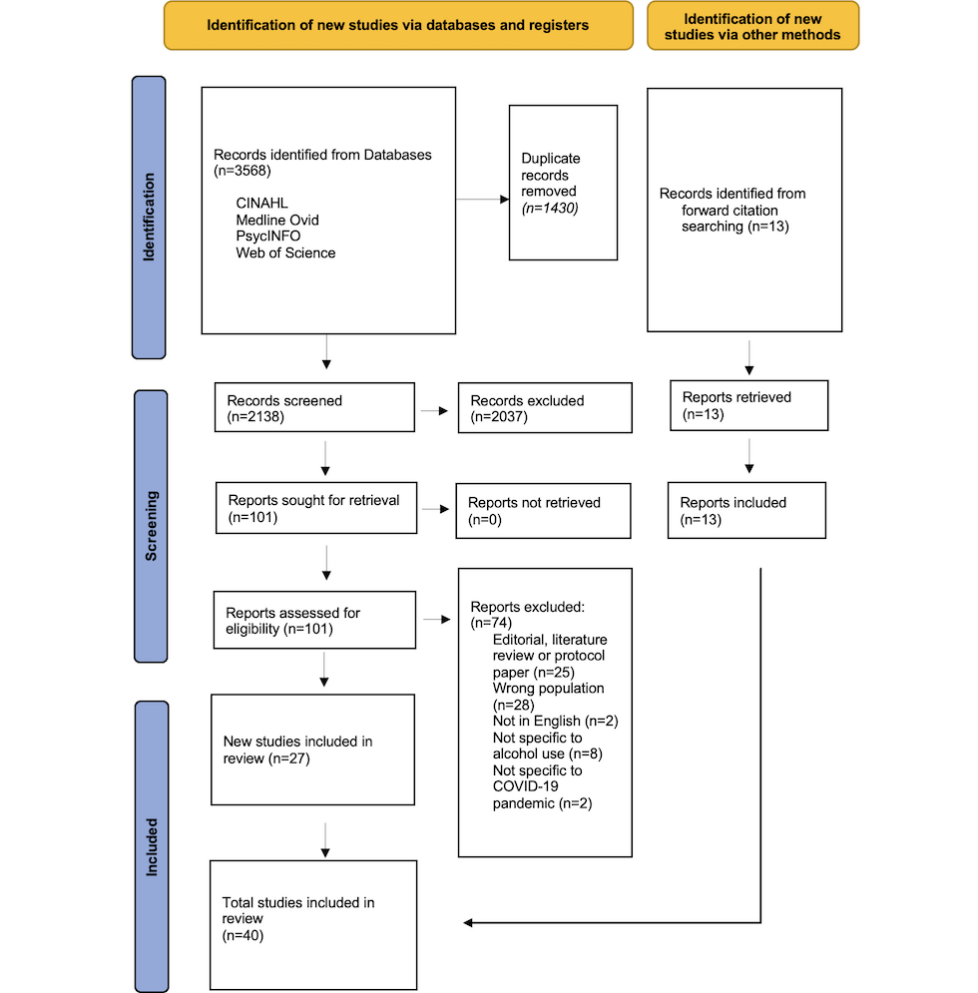
PRISMA-ScR (Preferred Reporting Items for Systematic Reviews and Meta-Analyses extension for Scoping Reviews) flow diagram.

After 1430 (40.08%) duplicates were removed from the initial pool of 3568 articles, 2138 (59.92%) articles were screened for eligibility in a 2-round process (Figure 1). In the first round, article titles and abstracts (and text as needed) were reviewed against the PCC framework. We included peer-reviewed empirical studies that were published in English with data collection occurring after the WHO declaration of the COVID-19 pandemic. Nonempirical papers; expert opinions; letters to the editor; preprints; and empirical papers that were published after March 11, 2020, but did not contain alcohol consumption data collected during the COVID-19 pandemic were excluded. Studies that included a comparison of alcohol use between households with children and households without children were included. Reasons for exclusion included study population not being parents with alcohol consumption (eg, adolescent alcohol use) and the lack of explicit data collection on alcohol consumption as a variable. Studies that collected data on adult alcohol consumption during the COVID-19 pandemic but did not differentiate between adults living with children at home and adults not living with children at home were also excluded. Most studies that remained included both parent and nonparent participants (30/40, 75%), while some studies examined parents specifically (10/40, 25%).

### Data Extraction

Data from the articles were extracted into an extraction table derived from Polit and Beck [20]. The table included columns for authors, country of origin, year of publication, research design, sample size, measurement of alcohol use, time frame of data collection, and main findings. Extraction was performed independently by team members (KB and KC) and checked for accuracy (KC). Concerns with data extraction were resolved in consultation with the first author (CO).

### Evidence Synthesis

A narrative analysis of the main findings was conducted to identify and compare themes across the included studies. This approach allowed for the evaluation and integration of diverse findings on the trends in parental alcohol consumption during the COVID-19 pandemic.

## Results

### Overview

Of the 40 studies included in analyses, 36 (90%) were quantitative studies, 2 (5%) were qualitative studies, and 2 (5%) were systematic reviews (Figure 1). Of the 36 quantitative studies, most studies (n=28, 78%) were cross-sectional survey studies, 7 (19%) studies were longitudinal cohort studies, and 1 (3%) was a secondary data analysis of publicly available data (Table 1). The following themes were identified: (1) having children at home was a factor associated with parental patterns of alcohol use, (2) mixed patterns of alcohol consumption among mothers and fathers, and (3) heterogenous linkages between parental patterns of alcohol use and mental health.

**Table 1:** Evidence extraction table.

Author and year		Time frame and country	Research design and sample size, N	Alcohol variable	Main findings
**Global**					
	Kyaw Hla et al [21], 2021	April 17 to June 25, 2020 Global	Cross-sectional survey N=37,206; n=28,649 (77%) women	Respondents were asked about alcohol “stock up” and consumption frequency compared to pre–COVID-19 levels.	Middle-aged, educated women working from home and living with children were a high-risk group for increased alcohol use during the lockdown. Households with children were more likely to increase alcohol use (ORa 1.17, 95% CI 1.10-1.24; P<.001) compared to households with adults only.
	Roberts et al [4], 2021	December 2019 to November 2020 Global	Systematic review N=45 articles	In the included studies, respondents were asked about patterns of alcohol use, harmful alcohol use, and binge drinking.	Of the 45 studies, 5 (11%) studies found significantly higher alcohol use among parents compared to nonparents.
	Sallie et al [22], 2020	May 12 to 28, 2020 Global	Cross-sectional survey N=1346; n=209 (15.53%) parents	Respondents were asked about drinking behaviors before and during COVID-19 lockdowns. 3 items from the AUDIT-Cb	Drinking behaviors decreased overall during quarantine across the entire sample. Among parents, drinking behaviors increased compared to nonparents (P=.003).
	Schmidt et al [23], 2021	March 2020 to March 2021 Data collection occurred between March and May 2020 in most articles (49/53, 92%). Global	Systematic review N=53 articles	Alcohol concept varied in the included studies. The PICOc tool was used to guide the search strategy where the problem identified was substance use, substance use disorder, drug abuse, and dependence.	Caregiving responsibilities were discussed as a factor in increased substance use in 13 (25%) of the 53 articles. Parental status was associated with higher overall alcohol consumption, higher Alcohol Use Disorders Identification Test scores, consuming more drinks per occasion, more drinks consumed per week, and more heavy drinking episodes, with a positive correlation between the number of children at home and the amount of alcohol consumed. One study found that having children at home was associated with decreased alcohol use, and another study found that having children at home was associated with less binge drinking.
**North America**					
	Deacon et al [24], 2021	July 2020; reported retrospectively in April 2020 Canada	Cross-sectional survey N=758 couples; n=211 (27.8%) homeschooling couples; n=173 (22.8%) couples homeschooling due to the COVID-19 pandemic	Brief Alcohol Motives Measure (2 items from the scale were used to examine coping-related alcohol use)	Among the homeschooling sample, coping-related alcohol use was significantly increased relative to prepandemic use. The partner effect of hours spent homeschooling was significant on coping-related drinking.
	DesRoches et al [25], 2021	April to July 2020 Canada	Cross-sectional survey N=758 couples; n=211 (27.8%) homeschooling couples	Quantity, frequency, and Peak Alcohol Use Index	Women’s hours of homeschooling were associated with greater drinking frequency in both the women themselves (beta=0.04; P=.01 and in their partner (beta=0.029; P=.02). Longer hours of homeschooling by men were associated with lower drinking frequency in women partners (b=–0.04; P=.02), but not with their own. Homeschooling was significantly correlated with drinking quantity (b=0.17; P<.05) but not drinking frequency. Partner’s time spent home schooling was positively related to one’s own drinking frequency (b=0.022; P=.01), quantity (b=0.020; P=.03), and peak drinking (b=0.022; P=.02). Gender significantly moderated the effect of time spent homeschooling on drinking frequency in dyadic analysis with both actor (P=.009) and partner (P<.001).
	Gadermann et al [26], 2021	May 14 to 29, 2020 Canada	Cross-sectional survey N=3000; n=618 (20.6%) parents	Respondents were asked about increased alcohol consumption after the pandemic as a means of coping with pandemic-related stress or deteriorating mental health. Questions were adapted from the Mental Health Foundation survey.	Parents had a significantly greater increase in alcohol consumption compared to nonparents (28% vs 16%; P<.001). This was greater among men (32% vs 24%; P=.01). Parents were more likely to report deteriorated mental health (44.3% vs 35.6%; P<.001).
	Hill MacEachern et al [27], 2021	September 11 to December 4, 2020 Canada	Cross-sectional survey N=12,344; n=3474 (28.14%) women and n=3348 (27.12%) men were parents or guardians to children aged <18 years	Respondents were asked, “how has your alcohol consumption changed since before the COVID-19 pandemic?”	Women with children were 1.46 times (95% CI 1.13-1.90) more likely to report increased alcohol consumption compared to women without children (23.3% vs 13.4%). Men with children were 1.38 times (95% CI 1.05-1.82) more likely to report increased consumption compared to men without children (21.7% vs 12.7%).
	Joyce et al [28], 2022	April 14 to 28, 2020 Canada	Cross-sectional survey N=508 mothers of children aged 0 to 8 years	Respondents were asked, “has your alcohol/drug use changed since the COVID-19 pandemic began?”	Among the participants, 54.9% (n=279) reported no change in substance use, 39.2% (n=199) reported increased substance use, and 5.9% (n=30) reported decreased substance use. Alcohol was the most commonly reported substance (406/508, 80%), followed by cannabis (44/508, 8.7%).
	Thomson et al [29], 2021	May 2020, September 2020, and January 2021 Canada	Multiround cross-sectional surveys May 2020: n=618 parents September 2020: n=804 parents January 2021: n=602 parents	Respondents were asked whether their alcohol consumption had changed as a result of the COVID-19 pandemic.	Parents were significantly more likely to report increased alcohol consumption compared to nonparents in all 3 rounds of surveys: round 1 (27.2% vs 16.1%), round 2 (21.9% vs 14.8%), and round 3 (22.4% vs 15.4%). Parents aged <35 years had higher odds of increased drinking than older parents (OR 1.51, 95% CI 1.10-2.07). Women were less likely than men to increase drinking (OR 0.76, 95% CI 0.59-0.97). Parents who continued to work while looking after children had higher odds of increased drinking (OR 1.86, 95% CI 1.45-2.40).
	Wardell et al [30], 2020	April to May 2020 Canada	Cross-sectional survey N=320; n=80 (25%) parents	Respondents were asked about their alcohol consumption in the past 30 days and the month before the COVID-19 pandemic. Questions were modified from those provided by the National Institute on Alcohol Abuse and Alcoholism.	Having a child in the household was a significant predictor of drinking as a coping behavior and drinking problems (β=.10; P<.05).
	Zajacova et al [31], 2020	March 29 to April 3, 2020 Canada	Secondary data analysis N=4319 respondents; 30% (n=1296) had children aged <18 years	Respondents were asked whether weekly alcohol consumption habits had changed.	Those with children were more likely to report a decrease in alcohol consumption (P<.05).
	Barbosa et al [32], 2023	February 2020, April 2020, July 2020, and November 2020 United States	Longitudinal survey design N=557; n=146 (26.3%) parents	Respondents were asked about the quantity and frequency of alcohol consumption and binge drinking.	The trajectory of alcohol consumption among parents found a 64% increase in the number of drinks consumed per month from February 2020 to November 2020. The increase in drinks consumed was significantly larger (P<.05) for those with children in the household than for those without children.
	Boschuetz et al [33], 2020	April 5 to 12, 2020 United States	Cross-sectional survey N=408; 83% (338/405) were women and 80% (303/404) had children at home	AUDIT-C	Having children at home was associated with a significant increase in AUDIT-C scores (P=.02).
	Freisthler and Price Wolf [34], 2023	April 2020, April 2021, and April 2022 Ohio, United States	3 longitudinal waves of surveys, during April 2020, 2021, and 2022 N=266 mothers across the 3 waves	Mothers were asked how often they drank any kind of alcoholic beverage in the past year, on how many of the past 28 days they had at least 1 drink, and how many drinks were consumed in the past 28 days.	Mothers reported fewer days of alcohol consumption in April 2021 and April 2022 compared to April 2020. However, the average number of drinks per day was higher in April 2021 and April 2022 than in April 2020. Mothers reported drinking less frequently but drinking more in volume when they did drink.
	Grossman et al [35], 2020	May 2020 United States	Cross-sectional survey N=832; 45.1% (n=375) had children aged <18 years at home	Respondents were asked about drinking frequency, binge drinking, patterns of drinking, and factors related to drinking in the past 30 days.	Those with children at home consumed alcohol on a greater number of days than those without children (13.0 days vs 11.6 days; P=.054). There were no significant differences between parents and nonparents in the total drinks consumed or binge drinking.
	Knell et al [36], 2020	April 15 to May 5, 2020 United States	Cross-sectional survey N=1804; n=785 (43.5%) parents	BRFSSd If alcohol use was reported, further questions were asked about the “average number of daily drinks” and whether this number changed since the COVID-19 pandemic.	Having children increased the odds of increased alcohol consumption (OR 1.58, 95% CI 1.19-2.09).
	Lamar et al [37], 2021	March 24 to April 28, 2020 United States	Cross-sectional survey N=1048 parents	Alcohol Use Disorders Identification Test	Problematic alcohol use was indicated among 26.5% (n=278) of the sample, with 11.4% (n=119) indicating a high level of problematic alcohol use. Male participants had significantly higher consumption compared to female participants (t1046=0.02; P=.003).
	Nordeck et al [38], 2022	March to July 2020 United States	Longitudinal study 5 waves starting from March 11, 2020 N=4298 across 5 surveys 29.3% (n=1259) living with children	The number of drinking days in the past 7 days	The number of drinking days was lower for participants living with a partner and children (β=–.65; 95% CI –0.82 to –0.48) and those living with children only (β=–.86; 95% CI –1.16 to –0.57) compared with participants without children. However, there were significant sustained increases in drinking days among those living with a partner and children compared to those living with children only or those in other household structures.
	Pomazal et al [39], 2023	May 2020 to August 2021 Wisconsin, United States	Longitudinal study (3 waves) Wave 1: n=1290 Wave 2: n=1868 Wave 3: n=1585 Percentage of participants with children at home at each wave: 29.4% (379/1290), 28.3% (528/1868), and 25.3% (401/1585), respectively.	Individuals were asked to self-report alcohol consumption in the last 60 days (a lot more, a little more, same, little less, or much less) compared to the reference period (prepandemic period, then July 2020, and February 2021)	In all 3 waves, the presence of children at home was associated with increased drinking (34.56%, 25.57%, and 22.38%; P<.001). Adjusted logistic regression model data: participants aged 55 years with children at home were less likely to increase drinking than those aged 35 years (wave 1: OR 0.23, 95% CI 0.1-0.53; wave 2: OR 0.4, 95% CI 0.17-0.91) and those aged 40 years (wave 1: OR 0.22, 95% CI 0.09-0.54; wave 2: OR 0.41, 95% CI 0.17-0.97) with children at home.
	Rodriguez et al [40], 2021	July 22 to August 4, 2020 United States	Cross-sectional survey N=118 couples n=100 (84.75%) couples with children at home	Respondents were asked about alcohol consumption patterns in the month before completing the survey. Items from Inventory of Problems–Alcohol and Drugs	Having children at home was a significant predictor for drinking to cope (2.45; P<.05; CI 3.3-31.6). No significant association between having children at home and drinks consumed per week, high-intensity drinking, or alcohol-related problems.
	Weerakoon et al [41], 2021	March to April 2020 United States	Cross-sectional survey N=1928; 42% (n-=810) had children living in the household	Respondents were asked about changes in drinking habits and binge drinking behaviors during the COVID-19 pandemic.	Households with children were less likely to binge drink than those without children (AORe 0.74, 95% CI 0.58-0.94). No significant changes were reported in parental drinking compared with participants with no children living at home.
**Australia**					
	Booth et al [42], 2024	September 2020 Australia	Cross-sectional survey N=4022	Respondents were asked to rank how often they drank alcohol before and during the lockdown on a scale from 1 (never) to 7 (≥2 times a day)	Those with children were more likely to experience an increase in alcohol consumption (β=.51, 95% CI 0.37-0.76).
	Callinan et al [43], 2021	April 29 to May 16, 2020 Australia	Cross-sectional survey N=2307; n=468 (20.3%) parents with dependent children	Respondents were asked about their 2019 drinking behaviors (prepandemic period) and drinking behaviors in the past 30 days.	Having dependent children was significantly associated with increased alcohol consumption (β=.62; 95% CI 0.32-0.92; P<.05), and homeschooling responsibilities were significantly associated with increased alcohol consumption (β=.53, 95% CI 0.2-0.82; P<.05).
	Cook et al [13], 2021	July to September 2020 Australia	Qualitative study N=30 parents and caregivers of children aged 4 to 12 years	Participants were asked about the nature of family lives before and after the COVID-19 pandemic, including changes in alcohol practices and family dynamics due to the COVID-19 pandemic.	Alcohol use was reported to signal the end of the workday as a means of self-care and to alleviate boredom and manage stress. It was associated with feelings of guilt due to lockdown challenges.
	Glenister et al [44], 2021	May 29 to July 9, 2020 Australia	Cross-sectional survey N=339 rural women; 41% (n=139) of sample lived with children	Respondents were asked whether alcohol use increased, decreased, or remained the same since the COVID-19 pandemic.	Rural women living with children were more likely to report increased alcohol consumption compared to rural women not living with children (OR 2.37, 95% CI 1.36-4.15; P=.002).
	Greenwood et al [45], 2023	April 2020 to May 2021 Victoria, Australia	Longitudinal wave-based surveys (13 waves) N=2261 parents	Parents were asked about the frequency of alcohol consumption per month (ranked on a 7-point scale).	Estimated alcohol frequency trajectory for parents showed a decreased use over the course of the pandemic. Female and other parent gender were associated with a trajectory of lower frequency of alcohol use. Older parent age was associated with a trajectory of higher frequency of alcohol use.
	Johnson et al [46], 2021	June to July 2020 Australia	Cross-sectional survey N=406 mothers	Alcohol Use Disorders Identification Test Respondents were asked how their drinking has been impacted by the COVID-19 pandemic.	Of the sample of 406 mothers, 54.9% (n=223) exceeded drinking guidelines and 41.4% (n=168) reported drinking more due to the pandemic. As parenting stress increased, alcohol use increased (P=.002).
	Westrupp et al [47], 2023	April 8 to 28, 2020 Australia	Cross-sectional survey N=2365 parents	Respondents were asked how often they drank alcoholic beverages.	Compared to prepandemic population-based data from 4 Australian data sets, parents reported more frequent alcohol consumption (333/2365, 14.1%, pandemic data set) reported drinking on ≥4 days per week vs 771/9764, 7.89% (pre-pandemic data set), 7.9% reported drinking before the pandemic; P<.001). Women were less likely than mento consume alcohol at higher levels.
**Europe**					
	Bramness et al [48], 2021	June to July 2020 Norway	Cross-sectional survey N=1328; of the n=1200 who reported any alcohol use, n=887 (66.79%) had children aged <18 years in the household	Respondents were asked to report alcohol use in the past 12 months and changes in alcohol use since the COVID-19 measures were implemented. Two items from AUDIT-C	Among the entire sample, 56.8% (n=754) reported no change in drinking patterns, 29.9% (n=397) reported less drinking, and 13.3% (n=177) reported more drinking. Having a child aged <18 years in the household was associated with more drinking (P=.02).
	Koeger et al [49], 2022	April 2020 to January 2021 Germany	Multiround cross-sectional surveys Parents: round 1, n=307; round 2, n=295; and round 3, n=285	Participants were asked about AUFf on a weekly basis.	Odds for an increased AUF was higher among participants with children in round 1 during the first lockdown (OR 1.34, 95% CI 0.92-1.96; P>.05) and in round 2 during the relaxation of lockdown restrictions (OR 1.77, 95% CI 1.118-2.65; P<.01).
	Mangot-Sala et al [50], 2022	April 2020 to July 2021 The Netherlands	Longitudinal survey design N=63,194	Respondents were asked how many glasses of alcohol were consumed in the past 7 days.	Results showed that during periods of lockdown, households with children reported the lowest alcohol consumption. During the summer, households with children reported a seasonal increase in drinking related to relaxed COVID-19 restrictions.
	McAloney-Kocaman et al [51], 2022	March to June 2020 United Kingdom	Longitudinal study (multiwave survey) N=1268; 468 (36.9%) respondents had children in the household	Participants were asked to indicate a change in alcohol consumption (drinking less, drinking approximately the same, or drinking more than usual) after the implementation of the lockdown.	Perceived changes in alcohol consumption were significantly associated with the presence of children at home (χ22=20.3, P<.001). Odds of increased alcohol consumption was lower for those with children in the household.
	Oldham et al [52], 2021	April 30 to June 14, 2020 United Kingdom	Cross-sectional survey N=2777 parents and nonparents	AUDIT-C	Among men, living with children was significantly associated with increases in the units of alcohol consumed per drinking session (OR 1.72, 95% CI 1.09-2.73; P=.02) and the frequency of heavy episodic drinking (OR 2.40, 95% CI 1.44-3.99; P=.001). There was no significant increase in drinking for women living with children.
	Thorell et al [53], 2022	April 28 to June 21, 2020 Sweden, Spain, Italy, United Kingdom, Belgium, Netherlands, and Germany	Cross-sectional survey N=6720 parents of children aged 5 to 18 years; n=5914 (88%) female	Respondent were asked how their alcohol or drug changed use during the COVID-19 pandemic compared to their prepandemic use.	Across the sample, 5% of homeschooling parents reported increased levels of alcohol or drug use compared to prepandemic use. Differences varied by country. In the United Kingdom, there was a 19.1% increase, whereas in Sweden, Spain, and Italy, <3% reported increased drinking or drug use.
	Vanderbruggen et al [54], 2020	April 9 to 29, 2020 Belgium	Cross-sectional survey N=3632; 44.3% (n=1609) lived with children	Respondents were asked about the average amount of alcohol consumed before and during the lockdown. Respondents were asked whether they drank more, less, or the same amount as before the COVID-19 pandemic.	Overall, 30.3% (n=1100) of the total sample reported increased alcohol consumption, while 13.7% (n=498) reported decreased consumption. Alcohol use was positively correlated with the number of children living at home (22% increase in odds with every child at home; OR 1.220, 95% CI 1.146-1.289).
	Villette et al [55], 2022	January to March 2021 Western Brittany, France	Cross-sectional descriptive survey N=2491	AUDIT-C questionnaire used to assess change in alcohol consumption before, during, and after the lockdown (frequency of alcohol consumption, number of drinks per day, and frequency of heavy drinking)	Of those living with family, 30.19% (468/1550; P<.001) experienced a greater increase in alcohol consumption than those living with adult roommates (21/135, 15.6%) and those living alone (89/395, 22.5%). Living with family was associated with increased alcohol consumption (OR 0.62, 95% CI 0.46-0.83; P<.001).
**Central and South America**					
	Garcia-Cerde et al, [56], 2021	May 22 to June 30, 2021 Latin America	Cross-sectional survey N=12,328; n=8136 (66%) female	Respondents were asked to report on alcohol behaviors, including how often alcohol consumption occurred with children present.	An overall decrease in drinking during the COVID-19 pandemic (77.5% vs 65%) was found, including drinking with children present. Regression model found that quarantining (β=3.81; 95% CI 2.61-5.02; P<.001), anxiety (β=.42; 95% CI 0.20-0.63; P<.001), and higher income were positively associated with drinking with children present.
**Asia**					
	Sugaya et al [57], 2021	June 15 to 20, 2021 Japan	Cross-sectional survey N=11,427; n=6388 (55.9%) parents	Alcohol Use Disorders Identification Test (Japanese version)	Those who answered “yes” to the “presence of child” had higher hazardous alcohol use (11.6% vs 9.5%), lower no-problem scores (81.2% vs 83.2%), and equal potential alcoholism scores (7.2%, respectively) compared to those who answered “no” (χ2=12.4; P=.002).

^a^OR: odds ratio.

**^b^**AUDIT-C: Alcohol Use Disorders Identification Test–Consumption.

^c^PICO: Population, Intervention, Comparison, Outcome.

^d^BRFSS: Behavioral Risk Factor Surveillance System.

^e^AOR: adjusted odds ratio.

^f^AUF: alcohol use frequency.

Of the 40 included studies, 11 (28%) were also included in the 2 systematic reviews. To prevent undue inflation of support for the themes identified, we cited only the original papers to support specific themes.

### Having Children at Home as a Factor Associated With Parental Patterns of Alcohol Use

Many of the studies demonstrated linkages between parental alcohol use and the presence of children at home. Many studies found that, compared to not having children aged <18 years at home, having children at home was a significant predictor for an increase in alcohol consumption during the COVID-19 pandemic in the United States [32-36,38-40], Canada [26,27,29,30], Australia [42-44], Norway [48], Belgium [54], Germany [49], France [55], the United Kingdom [51,53], and Japan [57]. In a global survey study of 1346 adults (half of the sample were from the United Kingdom and United States), having children at home was a significant factor for increases in alcohol consumption, as operationalized by Alcohol Use Disorders Identification Test scores [22]. Participants in a nationally representative US sample of 2-parent households sustained increases in drinking days over the first 4 months of the COVID-19 pandemic, compared with the pre–COVID-19 baseline [38]. These findings are consistent with the conclusion of a higher incidence of increased drinking by adults with children at home compared with adults with no children at home in 2 systematic reviews during the COVID-19 pandemic [4,23]. Moreover, a large-scale global cross-sectional study involving 37,206 participants across 38 countries found households with children were significantly more likely to increase alcohol use compared to households with adults only [21]. An Australian survey of 4022 adults found that participants with children at home were more likely to increase the frequency of consuming alcohol along with other unhealthy foods (eg, snacks and sugared beverages) [42].

On the contrary, some studies indicated patterns of decreased parental drinking during the pandemic. In the United States, Weerakoon et al [41] found that there was no significant increase or decrease in parental drinking compared with participants with no children living at home (n=1928, 42% parents); moreover, having children in the household was associated with a decreased risk of binge drinking. Similarly, in Canada, Zajacova et al [31] found that in a sample of 4319 adults (of whom, 30% (n=1296) had children aged <18 years at home), having children at home was associated with a lower rate of alcohol consumption. A 13-wave longitudinal study of Australian parents found that the frequency of alcohol use decreased over time, although there was no pre–COVID-19 consumption comparison [45]. In a large study of 35 Latin American countries (N=12,328), the majority of participants endorsed decreased drinking during the COVID-19 pandemic when compared with prepandemic levels of drinking [56].

Studies carried out in Europe indicate a mixture of findings. In a study of 63,194 adults in the Netherlands, Mangot-Sala et al [50] found that when comparing adults living with children at home to adults living without children at home and adults living alone, adults living with children drank less than the other 2 groups, suggesting that having children at home was protective in terms of drinking behaviors. Households with children reported a transient increase in drinking to prepandemic levels only during the summer months when restrictions were relaxed and families were more likely to gather, socialize, and engage in drinking with others [50]. In a European study of 6720 parents from (in the order of the largest number of participants per country) Germany, Sweden, Spain, Italy, Belgium, the United Kingdom, and the Netherlands, the proportion of parents who endorsed increased drinking during the COVID-19 pandemic (5%) were largely concentrated in the United Kingdom, with 19% (N=509) of UK parents reporting increases in drinking behaviors [53]. This contrasts with the findings of another UK-based longitudinal study, which found that having children at home was associated with lower odds of increased consumption [51]. A multiround cross-sectional study of adults in Germany found that participants who had children had higher odds of increased alcohol use frequency during the first lockdown (not significant) as well as during the easement of restrictions (significant) but lower odds during the second lockdown (not significant) [49].

In Australia, using a qualitative study of parents, Cook et al [13] found that while many parents in the sample indicated that they increased their frequency of alcohol consumption, some reported that they used the COVID-19 pandemic as an opportunity to lower their frequency of drinking through the absence of social opportunities for drinking.

### Mixed Patterns of Drinking Among Mothers and Fathers

Several studies indicated that fathers were more likely than mothers to increase drinking during the COVID-19 pandemic in North America [26,29,37], Australia [45,47], and the United Kingdom [52]. This is in contrast to a global study by Kyaw Hla et al [21], where they found middle-aged, educated women with children at home to be a high-risk group for increased alcohol use during lockdowns. Hill MacEachern et al [27] also identified that, among parent participants, women had slightly higher odds of reporting increases in alcohol consumption when compared with men. Homeschooling was a significant predictor for increased drinking during the pandemic in North America [24] and Australia [43]. Moreover, in a Canadian sample of parents, having to engage in homeschooling for children had an effect on maternal drinking. Desroches et al [25] found that mothers spent more time homeschooling than fathers and that both parents drank more when mothers spent more time homeschooling. Moreover, mothers drank less when fathers spent more time homeschooling [25]. Across their European sample (N=6720), Thorell et al [53] found that across subsamples from Sweden, Spain, Belgium, the Netherlands, Germany, Italy, and the United Kingdom, only 5% of homeschooling parents reported increased alcohol use. Freisthler and Price Wolf [34] investigated mothers’ drinking patterns in a longitudinal study of US mothers via 3 waves of data collection (springtime of 2020, 2021, and 2022). Mothers reported significantly more days of alcohol consumption in the first wave when compared with the second and third waves; however, the average number of drinks consumed during a drinking day was greater in waves 2 and 3 [34]. In the qualitative study by Cook et al [13], one of the participants described how her own drinking habits changed while homeschooling during the pandemic:

But then by mid-April, I was completely out of work and we were still homeschooling, then drinking started about lunchtime, like come on, it’s 12 o’clock, it’s 5 o’clock somewhere, right.

Woman, Queensland

While Thomson et al [29] did not specifically examine homeschooling, they found that looking after children while working from home was associated with higher odds of increased alcohol consumption.

### Heterogenous Linkages Between Parental Patterns of Alcohol Use and Mental Health

Several studies looked at alcohol use as a coping mechanism for stress. In Australia, Johnson et al [46] found that parenting stress modestly correlated with the Alcohol Use Disorders Identification Test scores. In Canada, using path analysis, Wardell et al [30] found that having children at home was associated with greater alcohol consumption as a method of coping with pandemic stressors.

The findings regarding the relationships between alcohol consumption and mental health were mixed. Lamar et al [37] found a significant correlation between mental health symptoms (stress, depression, and anxiety) and increases in alcohol consumption in the United States. Qualitatively, parents in Australia described that partaking in alcohol consumption at the end of the day delineated a shift from time spent on the care of children to time for self and served a means of self-medicating stress and anxiety [13]. Garcia-Cerde et al [56] found anxiety to be a weak predictor of drinking with children present in Latin American countries. Thomson et al [29] and Joyce et al [28], in their Canadian studies, did not find a significant link between increased drinking and mental health symptoms, although Joyce et al [28] reported that mothers with a history of a previously existing anxiety disorder or elevated anxiety symptoms were more likely to increase their substance use. Notably, an alcohol use tracking app (Habit Tracker) that collected data from 83 countries (the majority from the United Kingdom and the United States) found that although participants with children (209/1134, 18.4% of the sample) indicated significant increases in drinking severity based on their Alcohol Use Disorders Identification Test–Consumption scores, their levels of depression and anxiety were lower relative to adults with no children, suggesting a protective effect of having children at home [22].

## Discussion

### Principal Findings

The findings of this scoping review provide a broad examination of the patterns of parental alcohol consumption during the COVID-19 pandemic. The global patterns of parental alcohol use were heterogeneous and were influenced by the stage of COVID-19 data collection and sociocultural contexts. Most of the studies clustered around the first year and a half of the pandemic with regard to data collection. While lockdown measures were associated with increased frequency and quantity of alcohol consumption for some Western, industrialized countries, notably the United States, Canada, Australia, and the United Kingdom, a number of studies found patterns of decreased consumption. In Latin American and European Union countries especially, lockdown restrictions eliminated opportunities for socializing and drinking outside the home. Although these findings were mixed, there have been concerning reports of increased alcohol-related deaths; a recent Statistics Canada [58] report indicated an 18% increase in alcohol-related deaths during the COVID-19 pandemic when compared to previous years and that this was the largest change in alcohol-related deaths over the past 20 years. Similarly, in the United States, there were relative increases in deaths when comparing 2019 and 2020 rates of alcohol-related mortality [59], with childbearing-aged adults (ie, those aged 25 to 44 years) experiencing the largest increases. Angus et al [60] also found that the rate of alcohol-related deaths (characterized as “deaths of despair”) increased in the United Kingdom during the COVID-19 pandemic. However, these reports did not distinguish adults who were parenting and those who were not.

Although we had expected to find an association between income support and alcohol use, many of the included studies did not focus on income as a predictor of alcohol use, with the exceptions of the study by Westrupp et al [47], which found a modest association between financial deprivation and lower alcohol use in Australia, and the study by Garcia-Cerde et al [56], which found that individuals with a higher income were more likely to drink with children present. McAloney-Kocaman et al [51], Greenwood et al [45], and Nordeck et al [38] did not separate parents from nonparents when examining the relationship between income and alcohol use and similarly found that lower income predicted decreased use. This contrasts with findings from the United Kingdom, which identified that the most socioeconomically disadvantaged households increased their alcohol purchases more than the least disadvantaged households [61].

In our study, we found some differences in the gender-related patterns of parental drinking, with some findings suggesting that fathers were more likely to increase overall alcohol consumption. Nonetheless, having to homeschool their children contributed to mothers’ increases in drinking. Homeschooling and the provision of childcare during day care closures were responsibilities that fell disproportionately on mothers [62], with women also bearing greater employment-related consequences, such as the reduction of work hours and job loss related to childcare responsibilities [63]. The marketing of alcohol to mothers through social media was especially rampant (eg, through the use of hashtags such as #sendwine and slogans such as “from wifi to wine time #distance learning”), which calls into question the ethics of unmitigated marketing by the alcohol industry [10,14]. In the United States, gender-related differences in drinking behaviors have changed over time, with increases in rates of alcohol consumption in adult women [64]. Some studies indicate both mothers and fathers tend to decrease drinking during the transition to parenthood, with little difference between genders [65]. Meanwhile, other studies indicate that parenting children aged <1 year was associated with lower maternal drinking rates, whereas men’s drinking habits changed little in response to parenthood [66]. Looking at overall drinking habits, fathers have been shown to be less likely to abstain from alcohol than mothers and consume greater volumes of alcohol [67]. Age has also been shown to be a factor, with young fathers more likely to partake in risky drinking behaviors than young mothers [67]. A systematic review of population-level alcohol policy interventions indicated evidence of gender-related differences in the impact of and exposure to alcohol marketing and failure to provide gender-specific recommendations [68].

Beyond the risk to parents’ own health are the possible effects of parental alcohol consumption on children. Children are inevitably exposed to parental alcohol use in the home environment, and parents may even provide alcohol for older or adolescent children as a way to “safely” introduce children to alcohol in a parent-controlled home setting [69,70]. In the United States, Maggs et al [71] found that in 1 in 6 families, parents permitted their adolescents to drink in the home with the family during the COVID-19 pandemic, changing from prepandemic family practices of not permitting adolescent drinking at home. Evidence indicates that parents supplying even sips of alcohol to children carries increased risks of adverse alcohol outcomes, such as adolescent binge drinking, while parental supply of whole drinks was associated with higher odds of binge drinking, alcohol-related harms, and symptoms of dependence for teens [69]. Taken together, there is reason to continue to investigate parental patterns of alcohol consumption, given the range of negative short- and long-term health outcomes for children.

### Strengths and Limitations

Strengths of this scoping review include the systematic search process followed. The findings of this scoping review are limited by the heterogeneous ways in which alcohol consumption was measured and the fact that not all studies had data on patterns of consumption before the COVID-19 pandemic for comparison purposes. This review includes a large number of studies from early in the pandemic and hence does not provide the complete picture of the patterns of parental alcohol consumption from the beginning of the COVID-19 pandemic in 2020 to the removal of pandemic-related restrictions during 2022 and 2023. Moreover, although the included studies differentiated patterns of alcohol consumption among parenting adults and nonparenting adults, a limited number of studies differentiated drinking habits between genders in parenting adults, which limited the provision of a more granular examination of the association between gender and alcohol consumption habits in parents.

### Clinical Implications and Future Directions

Parental drinking can adversely affect children indirectly through parent preoccupation and the diversion of parental attention and supervision and directly through physical and verbal violence in the home setting and influence children’s later drinking habits [67,72]. In the 2023 *Canada’s Guidance on Alcohol and Health* [73] report, the Canadian Centre for Substance Use and Addiction indicated that consuming >2 standard drinks per week heightens risks for breast and colon cancers, heart disease, and stroke*,* with the consumption of >6 drinks per week representing high risk of harms. In line with these strong recommendations for reducing alcohol intake to decrease health and social risks, clinicians who work with families (eg, primary care providers, nurses, and social workers) can inquire about clients’ frequency and amount of alcohol consumption and counsel strategies for moderating or reducing consumption. The findings of this review suggest that parents caring for children are a population that requires more empirical investigation in relation to the amounts and frequency of alcohol consumption and problematic alcohol use. Gender–based data analysis of parental drinking behaviors is also important for informing interventions and policies to promote safe alcohol use among parents. Because parents effectively serve as role models for children, their drinking habits can influence children’s later drinking behaviors. It is important to better understand how to assist parents in moderating alcohol intake, given the risks of unmitigated alcohol consumption.

### Conclusions

Our scoping review indicated that the COVID-19 pandemic influenced patterns of parental alcohol consumption in different ways. Sociocultural influences contributed to determining whether having children at home was a protective or risk factor for alcohol consumption. In countries where drinking alcohol is more likely to occur in social settings, such as at bars or restaurants (eg, Latin American and some European countries), parental consumption tended to decrease during the lockdown restrictions, while in countries where drinking in the home environment is the norm (eg, the United States, Canada, the United Kingdom, and Australia), parental consumption tended to increase. Given the known harms of alcohol, clinicians can initiate conversations about parental drinking habits and counsel moderation for parents who report amounts of drinking higher than national guidelines.

## Appendix

Multimedia Appendix 1 Search strategy.

Multimedia Appendix 2 PRISMA-ScR (Preferred Reporting Items for Systematic Reviews and Meta-Analyses Extension for Scoping Reviews) checklist.

## Abbreviations

AUDIT: Alcohol Use Disorders Identification Test
PCC: Population, Concept, and Context
WHO: World Health Organization

## Data Availability

Full data extraction matrix is available upon request.
