# Supplementary material for: Parental Patterns of Alcohol Consumption During the COVID-19 Pandemic: Scoping Review
Source: Interact J Med Res. 2024 Aug 26;13:e48339. doi: 10.2196/48339 (PMC11384174; doi:10.2196/48339)
Supplement: Multimedia Appendix 1 [file ijmr_v13i1e48339_app1.docx]

Supplementary file: Search strategy

Development of search strategy involved:

- Define review objective and question.
- Determine databases for search.
- Develop strings of synonyms that capture core concepts.

Concept 1

Covid or Covid-19 or Coronavirus or “corona virus” or pandemic or epidemic or outbreak or lockdown or quarantine or restrictions or “social distancing” or “coronavirus disease 2019” or Sars-cov-2

Concept 2

Parents or carers or fathers or mothers or maternal or paternal or family or children or dependents or childcare or homeschooling or working from home or caregiver or infant or toddler or preschool or “pre-school” or “school aged” or “pre-adolescent” or “pre-teen”

Concept 3

“Alcohol drinking” or alcohol or “alcohol use” or “alcohol use disorder” or drinking or intoxicated or “alcohol consumption” or ETOH or “alcohol-related harms” or drunk or “alcohol intake”

Our search for literature involves both using controlled vocabulary and natural language. We first identify whether thesauri are available in databases and search using major subject headings and relevant subheadings. Terms that are not part of major or minor subject headings are also identified and searched.

Search Strategy and Returns

Database: Ovid MEDLINE

| # | Searches | Results |
| --- | --- | --- |
| 1 | Alcohol Drinking/ | 75198 |
| 2 | alcohol drinking.mp. | 79649 |
| 3 | Alcohol drinking/ or exp Binge Drinking | 76771 |
| 4 | Parents | 80494 |
| 5 | Parents.mp. | 238848 |
| 6 | parents/ or exp fathers/ or exp mothers/ or exp single parent/ or exp surrogate mothers/ | 141471 |
| 7 | COVID- 19/ | 228126 |
| 8 | COVID- 19.mp. | 351286 |
| 9 | (Parents or carers or fathers or mothers or maternal or paternal or family or children or dependents or postpartum or childcare or homeschooling or working from home or caregiver or infant or toddler or preschool or pre-school or school aged or pre-adolescent or pre-teen).mp. [mp=title, book title, abstract, original title, name of substance word, subject heading word, floating sub-heading word, keyword heading word, organism supplementary concept word, protocol supplementary concept word, rare disease supplementary concept word, unique identifier, synonyms, population supplementary concept word, anatomy supplementary concept word] | 3797791 |
| 10 | (covid-19 pandemic or covid or covid-19 or coronavirus or corona virus or pandemic or epidemic or outbreak or lockdown or quarantine or restrictions or social distancing or coronavirus disease 2019).mp. [mp=title, book title, abstract, original title, name of substance word, subject heading word, floating sub-heading word, keyword heading word, organism supplementary concept word, protocol supplementary concept word, rare disease supplementary concept word, unique identifier, synonyms, population supplementary concept word, anatomy supplementary concept word] | 608076 |
| 11 | (alcohol drinking or ETOH or alcohol drinking or intoxica* or alcohol consumption or alcohol intake).mp. [mp=title, book title, abstract, original title, name of substance word, subject heading word, floating sub-heading word, keyword heading word, organism supplementary concept word, protocol supplementary concept word, rare disease supplementary concept word, unique identifier, synonyms, population supplementary concept word, anatomy supplementary concept word] | 177858 |
| 12 | (social distanc* or lock downs or lockdowns or restric* or quarantin*).mp. [mp=title, book title, abstract, original title, name of substance word, subject heading word, floating sub-heading word, keyword heading word, organism supplementary concept word, protocol supplementary concept word, rare disease supplementary concept word, unique identifier, synonyms, population supplementary concept word, anatomy supplementary concept word] | 638754 |
| 13 | (remote work or work from home or homeschool* or working from home).mp. [mp=title, book title, abstract, original title, name of substance word, subject heading word, floating sub-heading word, keyword heading word, organism supplementary concept word, protocol supplementary concept word, rare disease supplementary concept word, unique identifier, synonyms, population supplementary concept word, anatomy supplementary concept word] | 3273 |
| 14 | 1 and 4 and 7 | 3 |
| 15 | 1 and 2 and 3 and 4 and 5 and 6 and 7 and 8 | 3 |
| 16 | 9 and 10 and 11 and 12 and 13 | 10 |

Database: CINAHL

| # | Searches | Results |
| --- | --- | --- |
| 1 | (MH “COVID-19+”) | 42060 |
| 2 | (MH "Alcohol Drinking+") | 35677 |
| 3 | (MH "Parents+") OR (MH "Adolescent Parents+") OR (MH "Expectant Parents+") OR (MH "Fathers+") OR (MH "Mothers+") OR (MH "Parents of Children with Disabilities") OR (MH "Single Parent") OR (MH "Co-Parents") OR (MH "Biological Parents") OR (MH "Adoptive Parents") | 116356 |
| 4 | 1 and 2 and 3 | 2 |
| 5 | Covid or Covid-19 or Coronavirus or “corona virus” or pandemic or epidemic or outbreak or “coronavirus disease 2019” | 200717 |
| 6 | lock down or lockdown or quarantine or restrictions or “social distancing” | 51967 |
| 7 | "Alcohol drinking” or alcohol or “alcohol use” or “alcohol use disorder” or drinking or intoxicated or “alcohol consumption” or ETOH or “alcohol-related harms” or drunk or “alcohol intake” | 121835 |
| 8 | Parents or careers or fathers or mothers or maternal or paternal or family or children or dependents or postpartum or childcare or homeschooling or working from home or caregiver or infant or toddler or preschool or “pre-school” or “school aged” or “pre-adolescent” or “pre-teen” | 1554500 |
| 9 | 5 and 6 and 7 and 8 | 86 |
| 10 | 5 and 7 and 8 | 579 |
| 11 | Limit search 10 to date range 2020 01 01-2022 07 31 | 194 |
| 12 | Limit search 9 to date range 2022 01 01 – 2022 07 31 | 60 |
| 13 | 5 or 6 or 7 or 8 | 1834321 |
| 14 | 1 or 2 or 3 | 192372 |

Database PsycInfo

| # | Searches | Results |
| --- | --- | --- |
| 1 | DE "COVID-19" | 23674 |
| 2 | DE "COVID-19" OR DE "Post-COVID-19 Conditions" OR DE "Coronavirus" OR DE "Disease Outbreaks" OR DE "Quarantine" | 26688 |
| 3 | DE "Parents" | 64924 |
| 4 | DE "Adoptive Parents" OR DE "Expectant Parents" OR DE "Expectant Fathers" OR DE "Expectant Mothers" OR DE "Fathers" OR DE "Adolescent Fathers" OR DE "Single Fathers" OR DE "Foster Parents" OR DE "Homosexual Parents" OR DE "Mothers" OR DE "Adolescent Mothers" OR DE "Primipara" OR DE "Single Mothers" OR DE "Parental Characteristics" OR DE "Parent Educational Background" OR DE "Parental Attitudes" OR DE "Parental Investment" OR DE "Parental Occupation" OR DE "Parental Role" OR DE "Parenting Style" OR DE "Permissive Parenting" OR DE "Single Parents" OR DE "Single Fathers" OR DE "Single Mothers" OR DE "Stepparents" OR DE "Surrogate Parents (Humans)" OR DE "Coparenting" OR DE "Parenting" OR DE "Childrearing Practices" OR DE "Coparenting" OR DE "Parent Child Communication" OR DE "Parent Child Relations" OR DE "Parental Involvement" OR DE "Parenthood Status" OR DE "Parenting Skills" OR DE "Parenting Style" OR DE "Spouses" OR DE "Husbands" OR DE "Wives" | 167893 |
| 5 | DE "Quarantine" | 1529 |
| 6 | DE "Social Isolation" | 13528 |
| 7 | DE "Alcohol Use" | 27214 |
| 8 | DE "Alcohol Abuse" OR DE "Binge Drinking" OR DE "Alcohol Intoxication" OR DE "Acute Alcohol Intoxication" OR DE "Chronic Alcohol Intoxication" OR DE "Alcohol Use" OR DE "Alcohol Use Attitudes" OR DE "Alcohol Use Disorder" OR DE "Alcoholic Beverages" OR DE "Beer" OR DE "Liquor" OR DE "Wine" OR DE "Alcoholism" OR DE "Alcohol Induced Psychotic Disorders" OR DE "Alcohols" OR DE "Ethanol" OR DE "Isoproterenol" OR DE "Methanol" | 100212 |
| 9 | 1 and 3 and 7 | 3 |
| 10 | 1 and 3 and 5 | 0 |
| 11 | 2 or 5 or 6 and 4 and 8 | 14899 |
| 12 | Covid or Covid-19 or Coronavirus or “corona virus” or pandemic or epidemic or outbreak or or “coronavirus disease 2019” | 51987 |
| 13 | (“Alcohol drinking” or alcohol or “alcohol use” or “alcohol use disorder” or drinking or intoxicated or “alcohol consumption” or ETOH or “alcohol-related harms” or drunk or “alcohol intake”) tx | 210069 |
| 14 | (lockdown or “lock down” or quarantine or restrictions or “social distancing” or “remote work” or “work from home”) tx | 32197 |
| 15 | (Parents or carers or fathers or mothers or maternal or paternal or family or children or dependents or postpartum or childcare or homeschooling or working from home or caregiver or infant or toddler or preschool or “pre-school” or “school aged” or “pre-adolescent” or “pre-teen”) tx | 1521678 |
| 16 | 12 and 13 and 14 and 15 | 104 |
| 17 | Search 16 limited to yr = 2020 – 2022 | 93 |
| 18 | (2 or 12) AND (3 OR 4 OR 15) AND (7 OR 8 OR 13) AND (5 OR 14) | 88 |

Database: Cochrane Database of Systematic Reviews

| # | Searches | Results |
| --- | --- | --- |
| 1 | (Covid or Covid-19 or Coronavirus or corona virus or pandemic or epidemic coronavirus disease 2019).mp. [mp=title, short title, abstract, full text, keywords, caption text] | 355 |
| 2 | (Parents or carers or fathers or mothers or maternal or paternal or family or children or dependents or postpartum or childcare or homeschooling or working from home or caregiver or infant or toddler or preschool or pre-school or school aged or pre-adolescent or pre-teen).mp. [mp=title, short title, abstract, full text, keywords, caption text] | 6679 |
| 3 | (Alcohol drinking or alcohol or alcohol intake).mp. [mp=title, short title, abstract, full text, keywords, caption text] | 1316 |
| 4 | ((binge drink or intoxicat* or drunk or alcohol) adj3 harm).mp. [mp=title, short title, abstract, full text, keywords, caption text] | 14 |
| 5 | (lockdown or lock down or quarantine or restrictions or social distan*).mp. [mp=title, short title, abstract, full text, keywords, caption text] | 5758 |
| 6 | 1 and 2 and 5 | 208 |
| 7 | 3 or 4 | 1316 |
| 8 | 1 and 2 and 5 and 7 | 31 |

Database: Web of Science Core Collection

| 1 | “Covid-19 Pandemic” OR Covid OR Covid-19 or Coronavirus or “corona virus” or pandemic or epidemic or outbreak or lockdown or quarantine or restrictions or “social distancing” or “coronavirus disease 2019” (All Fields) | 1149430 |
| --- | --- | --- |
| 2 | ALL=(lockdown or lock down or quarantine or restrictions or social distanc* or work from home or remote work or home school* or homeschool*) | 787643 |
| 3 | Drinking OR “Alcohol drinking” or alcohol or “alcohol use” or “alcohol use disorder” or drinking or intoxicated or “alcohol consumption” or ETOH or “alcohol-related harms” or drunk or “alcohol intake” (All Fields) | 887697 |
| 4 | Parenting OR Parents or carers or fathers or mothers or maternal or paternal or family or children or dependents or postpartum or childcare caregiver or infant or toddler or preschool or “pre-school” or “school aged” or “pre-adolescent” or “pre-teen” (All Fields) | 720189 |
| 5 | 1 and 3 and 4 | 4011 |
| 6 | 1 and 2 and 3 and 4 | 1972 |
| 7 | Search 6 updated to 2020 – 2022 | 563 |
